# Real‐Time Monitoring of the Antiseizure Drug Valproic Acid Using a Novel Point‐Of‐Care Mass Spectrometry

**DOI:** 10.1111/cns.70499

**Published:** 2025-07-03

**Authors:** Xinqi Fang, Junhan Wu, Yuan Hong, Jiexun Bu, Wenpeng Zhang, Zheng Ouyang, Wei Hua, Ying Mao

**Affiliations:** ^1^ Department of Neurosurgery, Huashan Hospital, Shanghai Medical College Fudan University Shanghai China; ^2^ National Center for Neurological Disorders Shanghai China; ^3^ Shanghai Key Laboratory of Brain Function and Restoration and Neural Regeneration Shanghai China; ^4^ Neurosurgical Institute of Fudan University Shanghai China; ^5^ PURSPEC Technology (China) Ltd. Suzhou Jiangsu China; ^6^ PURSPEC Technology (Beijing) Ltd. Beijing China; ^7^ State Key Laboratory of Precision Measurement Technology and Instruments, Department of Precision Instrument Tsinghua University Beijing China

**Keywords:** drug monitoring, epilepsy, glioma, mass spectrometry, point‐of‐care testing, valproic acid

## Abstract

**Aims:**

To improve the timeliness and accuracy of anti‐epilepsy drug monitoring, we developed a point‐of‐care mass spectrometry (PoC MS) for real‐time quantification of valproic acid (VPA), as a paradigm for optimizing perioperative management.

**Methods:**

The PoC MS integrates a miniature mass spectrometer, paper capillary spray, and selective ion isolation. A total of 119 blood samples were analyzed for VPA quantification. An equivalent calibration model was established with 12 paired serum and whole blood samples. Analytical performance was benchmarked against liquid chromatography‐mass spectrometry (LC–MS) and enzyme multiplied immunoassay technique (EMIT) with 50 whole blood samples. A validation cohort of 45 samples collected at different time points from 9 patients was analyzed to monitor perioperative VPA concentration dynamics.

**Results:**

Selective ion isolation could improve signal intensity by threefold and enable VPA quantification down to 10 μg/mL within 3 min, demonstrating a clear speed advantage over other methods. The calibration model showed excellent agreement between whole blood and serum concentrations (*R* ^2^ = 0.978). PoC MS measurements of VPA closely matched LC–MS (*r* = 0.990, bias = 0.16%) and EMIT (*r* = 0.988, bias = 5.4%). In clinic, VPA level varied by up to 15‐fold across patients, and bedside PoC MS could depict dynamic VPA concentration profiles, with intra‐patient fluctuations averaging 26%, thereby supporting drug monitoring and therapeutic decision‐making.

**Conclusion:**

PoC MS could enable rapid, accurate, and matrix‐tolerant bedside monitoring of VPA from whole blood, supporting personalized antiseizure therapy and real‐time clinical decision.

## Graphical abstract

We present a novel point‐of‐care mass spectrometry system enabling rapid, real‐time quantification of valproic acid directly from whole blood. This bedside platform delivers serum‐equivalent results within 3 min and captures pharmacokinetic variability perioperatively, supporting individualized antiseizure therapy and timely clinical decision‐making in neurosurgical patients.

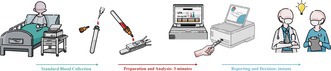

## 1 Introduction

Epilepsy is a prevalent neurological disorder characterized by a predisposition to recurrent, unprovoked seizures and affects over 50 million people worldwide [1]. It arises from diverse etiologies including genetic syndromes, trauma, stroke, and brain tumors [2, 3]. Particularly, seizures would affect up to 80% of lower‐grade gliomas (LGGs) [4], compared to 40% in glioblastoma (GBM) [5], with incidence increasing as the disease progresses [6]. Seizures are often the initial manifestation of glioma and significantly impact functional independence, quality of life, and long‐term neurological outcomes [7].

Tumor‐related epilepsy (TRE) arises from tumor‐host interactions that increase cortical excitability [8], which may involve neurotransmitter dysregulation, peritumoral inflammation, and glial network disruption in LGG [9]. In GBM, additional contributors include hypoxia, necrosis, blood‐brain barrier breakdown [7, 10], and enhanced electrical signaling activity at the cancer‐neuron interface [11]. Risk factors for TRE include younger age, IDH mutation, lower tumor grade, and cortical involvement, particularly in the frontal or temporal lobes [12, 13, 14]. Given the diverse and overlapping mechanisms underlying TRE, achieving adequate seizure control is particularly challenging yet crucial for preserving neurological function.

Pharmacological treatment is the cornerstone in the management of epilepsy. The International League Against Epilepsy (ILAE) and the American Epilepsy Society (AES) emphasize that antiseizure medication selection should consider the patient's broader clinical context, including tolerability, availability, and affordability, rather than efficacy alone [15, 16]. The European Association of Neuro‐Oncology (EANO) states that primary prophylaxis does not reduce seizure risks, emphasizing the need for timely, responsive therapeutic drug monitoring (TDM) [17]. Among available drugs, valproic acid (VPA) remains widely used in neurosurgical practice [8], owing to its broad‐spectrum efficacy, intravenous formulation, and long‐standing clinical familiarity [18, 19]. However, its use is complicated by a narrow therapeutic window, where excessive exposure may lead to hepatotoxicity, thrombocytopenia, hyperammonemia, and neurocognitive side effects [20], necessitating close TDM to maintain safe and effective dosage.

The pharmacokinetics of VPA are complex and highly variable. It is extensively protein‐bound (87%–95%) [21], hepatically metabolized via glucuronidation, β‐oxidation [22], and cytochrome P450 (CYP)‐mediated pathways [23], and exhibits low clearance (6–20 mL/h/kg) by hepatic metabolism [24]. Drug concentration is affected by genetic polymorphisms (e.g., UGTs and CYP2C9), hepatic function, albumin concentration, co‐medications, and perioperative physiological changes, which limit the reliability of fixed‐dose regimens and highlight the clinical necessity of individualized TDM [13].

Despite the importance of TDM, current methods are poorly suited for real‐time clinical decision‐making. Liquid chromatography‐mass spectrometry (LC–MS), while highly accurate, is time‐consuming and depends on centralized laboratory processing [25]. Immunoassays provide faster results, yet they require serum separation and lose reliability outside calibration range. Emerging electrochemical sensors hold promise for decentralized TDM but remain at the proof‐of‐concept stage without clinical deployment [26]. Other techniques, including desorption electrospray ionization (DESI) [27], direct analysis in real time (DART) [28], and paper spray ionization (PSI) [29], allow rapid analysis from minimally processed samples, but their reliance on bulky mass spectrometers constrains bedside integration.

We previously developed a miniature mass spectrometer (mini‐MS) for intraoperative IDH mutation detection [30], demonstrating its feasibility for real‐time neurosurgical applications. Building on this platform, we developed a novel point‐of‐care mass spectrometry (PoC MS) system for bedside TDM of antiseizure medications. The PoC MS is an all‐in‐one, battery‐powered unit that is compact enough for bedside use and requires no pumps, gas cylinders, or mains electricity. By combining ambient ionization, selective ion isolation, and a built‐in whole blood‐to‐serum calibration model, it delivers quantitative results in 3 min directly from just 100 μL blood sample without pretreatment. This study introduces a scalable framework for real‐time, individualized TDM method, with VPA serving as a proof‐of‐concept and the method readily extendable to other therapeutic agents.

## 2 Materials and Methods

### 2.1 Clinical Cohorts and Sample Collection

Three cohorts of adult glioma patients were studied at the Department of Neurosurgery, Huashan Hospital, Fudan University, with ethics approval (IRB KY2019‐587) and informed consent. Blood samples were exclusively obtained from residual complete blood count (CBC) specimens, eliminating the need for additional phlebotomy or disruption of clinical care. A total of 119 blood samples were analyzed for VPA quantification across the study.

For analytical evaluation and method comparison, 50 perioperative whole blood samples were collected and analyzed in parallel using liquid chromatography‐mass spectrometry (LC–MS), enzyme multiplied immunoassay technique (EMIT), and PoC MS. To establish a serum‐equivalent calibration model, 12 paired whole blood and serum samples were collected from glioma patients undergoing VPA therapy. For longitudinal monitoring, 9 patients were enrolled, and 45 serial blood samples were collected at predefined perioperative time points: within 3 days of admission, preoperatively, within 3 days postoperatively, on postoperative day 7, and prior to discharge. Each measurement was performed using 100 μL of whole blood per test.

### 2.2 Reagents and Instrumentation

Valproic acid (VPA) and its deuterated internal standard (VPA‐d_6_) were purchased from Aladdin Reagent Co. Ltd. and Pufen Biotechnology Co. Ltd. (Shanghai, China), respectively. HPLC‐grade acetonitrile was obtained from Thermo Fisher Scientific (NJ, USA), and distilled water (Watsons, China) was used throughout. The miniature mass spectrometer (mini‐MS) was provided by PURSPEC Technologies Ltd. (Suzhou, China). The mini‐MS includes a linear ion trap analyzer, a discontinuous atmospheric pressure interface (DAPI), and a paper capillary spray (PCS) cartridge. The device measures 33 × 23 × 15 cm, weighs ~8 kg, and operates at ~80 W. The DAPI opening was controlled via a 24 V DC pulse. The ion trap operated at a radio frequency of 1033 kHz, with resonance ejection at 376 kHz.

### 2.3 Ion Isolation via Stored Waveform Inverse Fourier Transform

Selective ion isolation was implemented using the stored waveform inverse Fourier transform (SWIFT) function integrated into the PoC MS system. A digitally synthesized excitation waveform was generated to selectively retain VPA (m/z 143) and its stable isotope‐labeled internal standard VPA‐d_6_ (m/z 149) within the ion trap. A dual‐notch filter was constructed in the frequency domain to define the isolation range, followed by inverse Fourier transformation to obtain the corresponding time‐domain waveform. Phase modulation was applied to suppress spectral side lobes and reduce off‐target ion excitation. The final waveform was amplified and applied to the trap electrodes, with an excitation duration of approximately 50–100 ms.

### 2.4 PoC MS Method for VPA Quantification

Exactly 100 μL of whole blood is mixed with 400 μL acetonitrile containing 40 μg/mL VPA‐d_6_ internal standard to precipitate proteins. After 30 s of vortex‐mixing, 100 μL of the supernatant is dispensed onto a disposable paper capillary spray (PCS) cartridge (PURSPEC Technologies Ltd.). The cartridge integrates a capillary tip combined with a paper‐based filter that pre‐processes the blood extract. The tip simultaneously serves as the electrospray emitter, enabling direct ionization without additional sample handling. Once the cartridge is inserted into the PoC MS system, ions are accumulated in the linear ion trap for 60 ms by synchronously closing the DAPI valve and activating the RF field. A dual‐notch SWIFT waveform then isolates the target ions of VPA (m/z 143) and its isotope‐labeled internal standard VPA‐d_6_ (m/z 149). Quantification is achieved by isotope‐dilution, using the peak‐height ratio of the two target ions against a five‐point calibration curve (10–150 μg/mL). The total analysis time is approximately 3 min per sample.

### 2.5 Reference Methods for VPA Quantification

VPA concentrations were determined using two established reference methods: liquid chromatography‐mass spectrometry (LC–MS) and enzyme‐multiplied immunoassay technique (EMIT). For LC–MS analysis, 50 μL of sample, calibrator, or quality control material was mixed with 100 μL of VPA‐d_6_, vortexed at 2000 rpm for 30 s, and centrifuged at 10,000 rpm for 5 min. A 110 μL aliquot of the supernatant was transferred to a new tube and centrifuged again at 4000 rpm for 3 min. A 1–5 μL volume of the resulting supernatant was injected into a reversed‐phase C18 chromatographic column. Detection was performed using electrospray ionization in multiple reaction monitoring mode, and quantification was based on a standard curve constructed with isotope‐labeled internal standard. For EMIT‐based immunoassay (Siemens), samples were analyzed on a fully automated biochemical analyzer (V‐Twin/Viva series) following the manufacturer's instructions. The assay employs a competitive binding reaction between endogenous VPA and an enzyme‐labeled antigen for a monoclonal antibody. Absorbance change at 340 nm was monitored to quantify VPA concentrations against a six‐point calibration curve (0–150 μg/mL). Samples exceeding the upper range were diluted and reanalyzed. Daily quality control procedures were performed using manufacturer‐provided standards.

### 2.6 Statistical Analysis

Raw MS data were acquired with PMS Client Pro (PURSPEC Technologies, Beijing, China) and pre‐processed in MATLAB 2019a (MathWorks, USA) for baseline correction and peak alignment. Statistical analyses were performed in GraphPad Prism 9 (GraphPad Software, USA). Data normality was assessed with the Shapiro–Wilk test (*α* = 0.05) and equality of variance with Levene's test. For two‐group comparisons, normally distributed data with equal variances were analyzed by an unpaired two‐tailed t‐test, whereas normal data with unequal variances were analyzed by Welch's *t*‐test. Datasets failing normality were compared with the Mann–Whitney *U*‐test (unpaired). Pearson's correlation was used when both variables were normal; otherwise, Spearman's rank correlation was applied. Bland–Altman analysis evaluated method agreement, and linear regression was used for whole‐blood/serum calibration; heteroscedasticity of residuals was checked with the Breusch‐Pagan test. Exact tests and *p*‐values are reported in the figure legends. Time‐concentration waterfall plots were generated in Python 3.9 (pandas, NumPy, matplotlib). A two‐sided *p* < 0.05 was considered statistically significant.

## 3 Results

### 3.1 Ion Isolation With SWIFT Would Improve Detection Limits and Quantitative Stability

**FIGURE 1:**
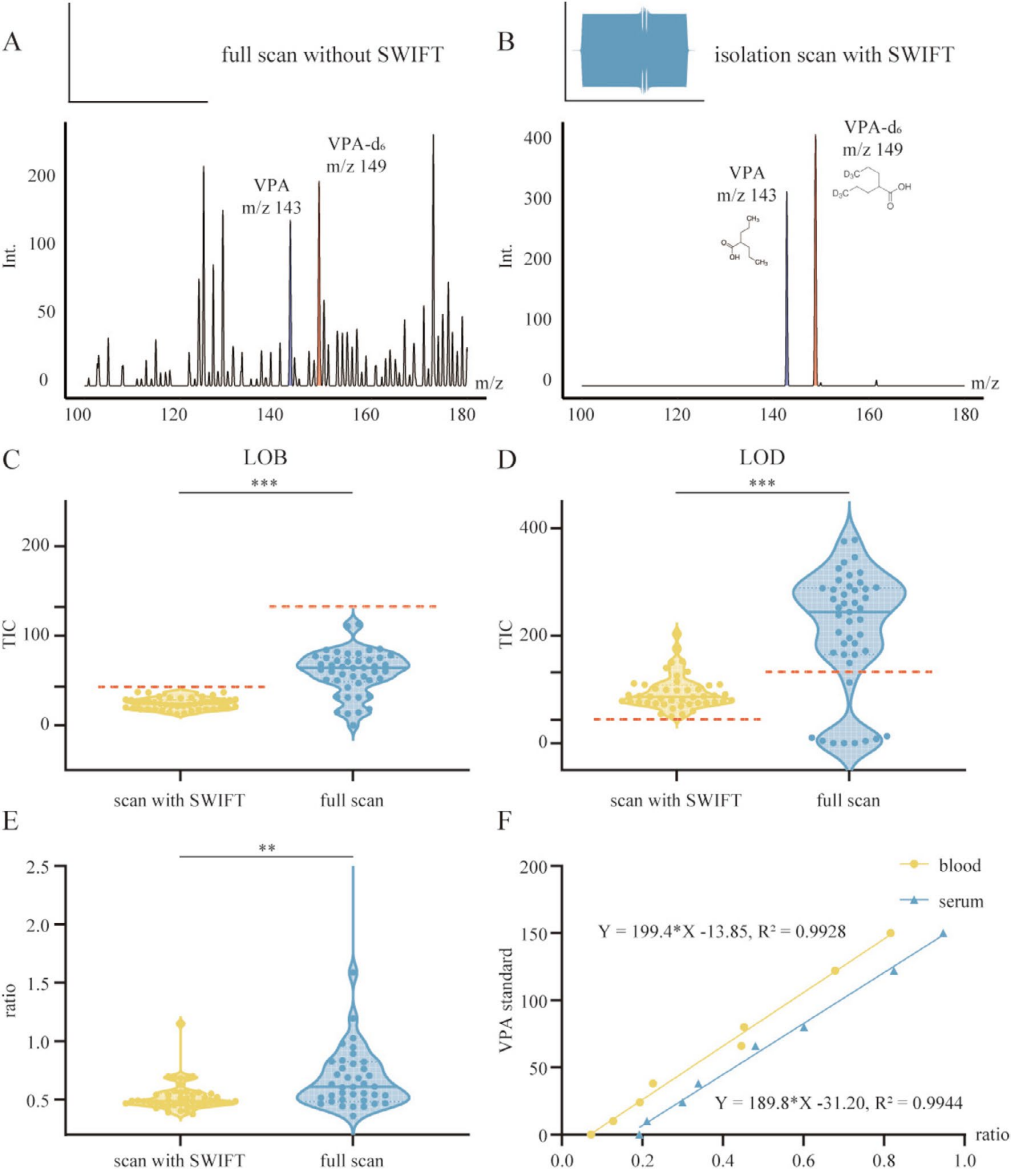
SWIFT scanning enhances sensitivity, precision, and linearity for VPA detection in whole blood. (A, B) Mass spectra of whole blood spiked with VPA and VPA‐d_6_ acquired using full scan and SWIFT mode. SWIFT selectively isolated target ions and improved the signal‐to‐noise ratio. (C) Background signal in VPA‐free replicates (*n* = 45) was significantly lower with SWIFT (24.2 ± 6.4) than with full scan (59.2 ± 24.4); unpaired t‐test with Welch's correction, *p* < 0.001. Derived LOD thresholds were 43.4 TIC (SWIFT) and 132.5 TIC (full scan) (red dashed lines). (D) At their respective detection thresholds (10 μg/mL for SWIFT; 38 μg/mL for full scan), SWIFT showed tighter signal dispersion (94.5 ± 31.7 vs. 210.1 ± 112.8 TIC; CV 33.5% vs 53.7%); Mann–Whitney *U*‐test, *p* < 0.001. (E) SWIFT produced a narrower VPA/VPA‐d_6_ ion‐ratio distribution (CV = 24.0%) than full scan (CV = 176%); Mann–Whitney *U*‐test, **p = 0.0021. (F) Calibration curves in whole blood and serum (10–150 μg/mL) showed strong linearity (*R* ^2^ = 0.993 and 0.994, respectively). **p < 0.01; ***p < 0.001.

The isolation scanning mode with stored waveform inverse Fourier transform (SWIFT) substantially improved VPA detection sensitivity in complex whole blood matrices by enabling high‐resolution isolation of target ions and effectively suppressing background interference from endogenous matrix components. In full scan, spectra were dominated by background ions, with VPA (m/z 143) and its internal standard VPA‐d_6_ (m/z 149) barely detectable above noise (Figure 1A). In contrast, scan with SWIFT selectively retained only the analyte and internal standard peaks, increasing their signal intensities by approximately threefold (Figure 1B).

To evaluate analytical performance, we compared the limit of blank (LOB) and limit of detection (LOD) between SWIFT and full scan using VPA‐free and low‐concentration samples. As expected, background signal intensity in blank replicates (*n* = 45) was significantly lower with SWIFT (*p* < 0.001, unpaired *t*‐test with Welch's correction), yielding a mean total ion count (TIC) of 24.2 ± 6.4 (CV: 26.6%) compared to 59.2 ± 24.4 (CV: 41.2%) in full scan. Applying the mean + 3SD criterion, the estimated LOD thresholds were 43.4 TIC for SWIFT and 132.5 TIC for full scan, with SWIFT exhibiting a markedly lower detection baseline (Figure 1C, red dashed lines). At each method's detection threshold—10 μg/mL for SWIFT and 38 μg/mL for full scan—SWIFT demonstrated improved precision and reduced signal dispersion, with a mean TIC of 94.5 ± 31.7 (CV: 33.5%) versus 210.1 ± 112.8 (CV: 53.7%) in full scan (*p* < 0.001, Mann–Whitney *U*‐test; Figure 1D).

Quantitative reproducibility was also improved under SWIFT. As shown in Figure 1E, the VPA/VPA‐d_6_ ion ratio exhibited a mean of 0.524 and a standard deviation of 0.126 (CV: 24.0%) across 45 replicates. In comparison, full‐scan measurements showed broader variability (mean: 0.963, SD: 1.7, CV: 176%), with an approximately 14‐fold wider range (10.9 vs. 0.774). This difference was statistically significant (*p* = 0.0021, Mann–Whitney *U*‐test; Figure 1E).

Calibration curves were established for both whole blood and serum across a clinically relevant range (10–150 μg/mL, Table S1), encompassing the therapeutic window of VPA. Both matrices exhibited excellent linearity over the tested concentration range, with correlation coefficients of 0.993 for whole blood and 0.994 for serum (Figure 1F). Direct quantification in whole blood closely aligned with serum‐based results, with only a modest difference in slope.

Collectively, these findings demonstrate that SWIFT scanning could provide a robust analytical foundation for accurate VPA quantification in complex blood matrices.

### 3.2 PoC MS Could Enable Serum‐Equivalent Modeling From Whole Blood for VPA Measurement

**FIGURE 2:**
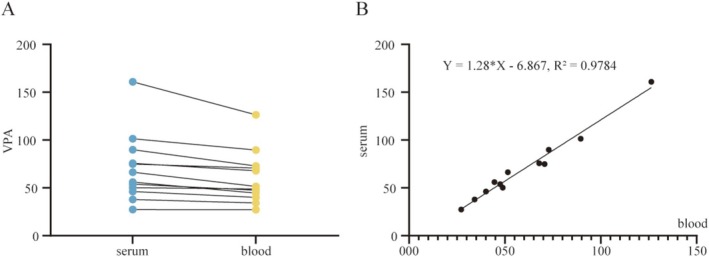
High correlation between VPA concentrations in whole blood and serum. (A) Paired measurements (*n* = 12) showed consistent agreement across matrices. (B) Regression analysis yielded *Y* = 1.28X–6.87 (*R* ^2^ = 0.978), supporting serum‐equivalent interpretation from whole blood results.

To enable serum‐equivalent interpretation using whole blood, we analyzed 12 paired perioperative whole blood and serum samples (Table S2). As shown in Figure 2A, VPA concentrations directly quantified from whole blood averaged 60.1 ± 27.5 μg/mL (range: 27.2–126.4 μg/mL, CV: 50.8%), while corresponding serum concentrations averaged 70.1 ± 35.6 μg/mL (range: 27.4–160.8 μg/mL, CV: 45.8%). Data points from the two matrices formed a dense, diagonally aligned cluster, visually confirming a strong correspondence across the clinically relevant therapeutic window (50–100 μg/mL and adjacent values).

Linear regression analysis revealed a highly significant correlation between whole blood and serum concentrations, with a Pearson correlation coefficient of 0.989 (95% CI: 0.961–0.997), and *p* < 0.001 (Figure 2B). The resulting equation, *Y* = 1.28X–6.87, suggests that whole blood concentrations require proportional adjustment for serum‐equivalent estimation. The slope of 1.28 (95% CI: 1.15–1.41) is consistent with the theoretical blood‐to‐plasma distribution ratio of VPA, while the negative intercept (−6.87 μg/mL) may reflect minor adsorption of VPA to cellular components. This systematic bias is automatically corrected by the device's embedded algorithm, minimizing the average absolute error in concentration conversion.

Model robustness was confirmed by Breusch‐Pagan testing (*χ* ^2^ = 1.02, *p* = 0.31), indicating homoscedasticity and the absence of significant outliers. These results validate the reliability of the whole blood‐to‐serum mapping approach and support its clinical applicability for interpreting VPA levels within the context of conventional serum‐based therapeutic thresholds.

### 3.3 PoC MS Might Offer a Rapid and Reliable Alternative to Conventional Methods

**FIGURE 3:**
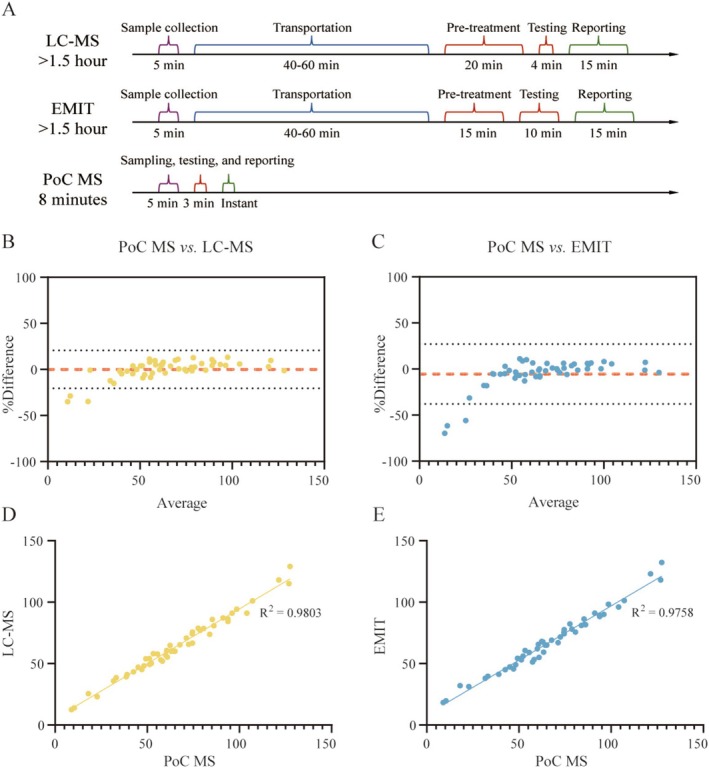
Workflow efficiency and analytical agreement of PoC MS compared with standard methods. (A) Turnaround time comparison showing that PoC MS enabled bedside VPA quantification within 8 min (including 5‐min sampling), while LC–MS and EMIT typically required ≥ 90 min due to transport and sample preparation delays. (B, C) Bland–Altman plots showing agreement between PoC MS and LC–MS (B) and EMIT (C); most deviations fell within ±20%. (D, E) Correlation plots demonstrating strong concordance (*r* = 0.990 vs. LC–MS; *r* = 0.988 vs. EMIT).

The turnaround time for VPA quantification was compared across three methods: LC–MS, immunoassay (EMIT), and PoC MS (Figure 3A). LC–MS, though highly sensitive, involved multiple sequential steps—including sample transport, serum preparation, instrument runtime, and report generation—resulting in a total turnaround time typically exceeding 90 min, and often reaching 1.5 to 2 h in routine clinical workflows. EMIT reduced some preparation steps but still required serum separation and centralized processing, with total turnaround times of approximately 90 to 110 min. In contrast, the full workflow with PoC MS took about 8 min, including 3 min for analysis and 5 min for standard blood collection (100 μL whole blood), as it eliminated transport and pretreatment steps, enabling direct bedside analysis without centrifugation or extraction.

To evaluate analytical performance, a method comparison study was conducted using a retrospective cohort of 50 whole blood samples from glioma patients receiving VPA administration (Table S3). Each sample was analyzed once using LC–MS (gold standard) and in triplicate by both EMIT and PoC MS. The measured mean VPA concentrations were consistent across platforms: 64.2 ± 24.8 μg/mL for LC–MS (range: 12.6–129.0 μg/mL; CV: 38.6%), 66.9 ± 24.6 μg/mL for EMIT (range: 18.3–132.3 μg/mL; CV: 36.8%; intra‐assay CV: 4.5%), and 66.0 ± 27.5 μg/mL for PoC MS (range: 8.9–118.6 μg/mL; CV: 41.7%; intra‐assay CV: 8.2%).

Bland–Altman analysis revealed good agreement between PoC MS and reference methods. Compared to LC–MS, PoC MS showed a mean bias of +0.16%, with 95% limits of agreement spanning −20.5% to +20.8% (Figure 3B). Relative to EMIT, the median bias was −5.4%, with limits ranging from −37.9% to +27.2% (Figure 3C). Notably, most deviations were observed at very low concentrations (< 20 μg/mL), outside the therapeutic range. Within the clinically relevant window (50–100 μg/mL ± 20 μg/mL), discrepancies were minimal (< 1%), indicating excellent agreement in the most critical decision‐making range.

Correlation analysis further confirmed analytical consistency. PoC MS exhibited a Pearson correlation coefficient of 0.990 (95% CI: 0.983–0.994; *R* ^2^ = 0.980) versus LC–MS (Figure 3D), and 0.988 (95% CI: 0.979–0.993; *R* ^2^ = 0.976) versus EMIT (Figure 3E), with scatter plots tightly clustered around the line of identity, affirming high linearity and comparability across methods.

### 3.4 PoC MS Could Capture Dynamic Pharmacokinetics and Support Individualized Dosing

**FIGURE 4:**
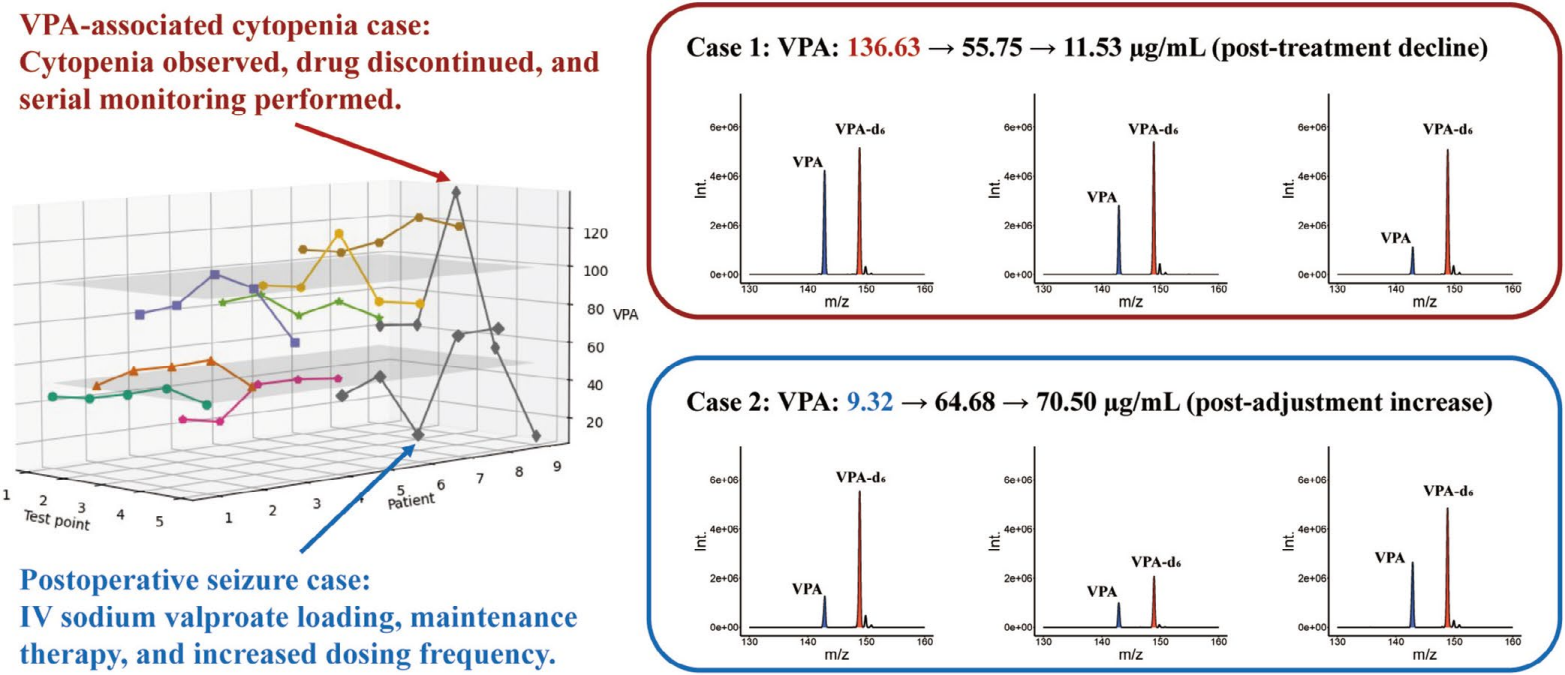
Clinical utility of real‐time PoC‐MS monitoring in perioperative VPA management: Early detection of adverse drug reactions and subtherapeutic levels guiding dose interventions. Red box: A postoperative patient with new‐onset cytopenia showed a supratherapeutic VPA level (136.6 μg/mL); treatment was withheld, and levels declined progressively to 11.5 μg/mL. Blue box: A patient with postoperative seizure had a subtherapeutic VPA level (9.3 μg/mL); an intravenous loading dose was administered, increasing levels to 70.5 μg/mL and resolving symptoms.

In a prospective cohort, nine glioma patients undergoing perioperative VPA administration were monitored using PoC MS at five key time points (Figure 4): within 3 days of admission, preoperatively, within 3 days postoperatively, on postoperative day 7, and prior to discharge. Despite receiving standardized VPA dosing, substantial pharmacokinetic variability was observed (Table S4). The mean VPA concentration across all patients and time points was 70.7 ± 15.5 μg/mL. Intra‐patient fluctuation averaged 26.0% (range: 4.9%–68.5%), and inter‐patient differences reached over 15‐fold, with postoperative levels ranging from 9.3 to 136.6 μg/mL.

To illustrate the clinical value of real‐time monitoring, two representative perioperative scenarios were analyzed. PoC MS detected a markedly elevated VPA concentration of 136.6 μg/mL on postoperative day 3 in a glioma case, coinciding with new‐onset cytopenia (platelets: 52 × 10^9^/L, WBC: 1.8 × 10^9^/L, Figure 4, red). Suspecting VPA‐related bone marrow suppression, therapy was promptly discontinued and supportive measures initiated. Follow‐up measurements confirmed a progressive decline in VPA levels (from 136.6 to 55.8, and to 11.5 μg/mL), accompanied by hematologic recovery (platelets: 112 × 10^9^/L).

PoC MS also revealed a subtherapeutic VPA level of 9.3 μg/mL (Figure 4, blue) in a patient who experienced a generalized seizure on postoperative day 2 despite standard dosing, prompting immediate dose adjustment, including an intravenous loading dose and increased administration frequency. Subsequent monitoring showed effective VPA titration, with concentrations rising to 64.7 and 70.5 μg/mL on postoperative day 7 and prior to discharge, respectively, and no further seizure events were observed.

## 4 Discussion

In this study, we developed an innovative bedside mass spectrometry (PoC MS) integrating a miniature mass spectrometer (mini‐MS), paper capillary spray ionization, and isolation scanning with SWIFT. This system could enable rapid and precise monitoring of VPA directly from whole blood samples. The real‐time quantitative PoC MS was validated for antiepileptic drug monitoring in a neurosurgical setting.

Accurate perioperative monitoring of antiepileptic drug concentrations is both challenging and clinically critical for glioma patients due to significant pharmacokinetic variability [31]. Our bedside measurements confirmed substantial intra‐ and inter‐patient variability despite standardized dosing. For instance, postoperative VPA levels varied widely among patients, ranging from subtherapeutic (9 μg/mL) to potentially toxic levels (136 μg/mL). Timely identification of these extreme concentrations using PoC MS could enable immediate clinical interventions, such as dosage adjustments or medication cessation. Real‐time therapeutic feedback would enable a shift from empirical dosing to proactive, individualized therapy, potentially improving neurological outcomes and reducing adverse effects [32].

Recent advances in mass spectrometry have expanded its clinical potential. Ambient ionization approaches, such as paper spray ionization [29], have facilitated rapid analysis of therapeutic drugs, metabolites, and bioactive species in biofluids with minimal sample preparation [33, 34]. These user‐friendly innovations have laid the groundwork for point‐of‐care diagnostics. However, widespread clinical adoption remains limited, largely due to the size, complexity, and operational demands of conventional mass spectrometry systems.

Methodologically, our study integrated several technological advances to overcome barriers traditionally limiting clinical TDM implementation. The mini‐MS enabled true bedside portability, paper capillary spray ionization allowed rapid whole‐blood analysis without pre‐processing, and SWIFT isolation scanning selectively isolated VPA ions, significantly enhancing detection sensitivity and analytical precision compared to traditional full‐scan acquisition. Specifically, SWIFT reduced interference from high‐abundance background ions, achieving approximately a threefold increase in signal intensity and a substantial reduction in analytical variability. These improvements enabled robust, low‐level quantification necessary for detecting subtherapeutic VPA levels critical to clinical decision‐making.

Our results demonstrated excellent analytical concordance between PoC MS and conventional laboratory‐based methods, including LC–MS and EMIT. Bland–Altman analysis confirmed minimal bias across clinically relevant concentration, indicating that PoC MS provides accuracy comparable to established laboratory techniques. The most significant advantage of PoC MS is its dramatic reduction in turnaround time: bedside analysis delivers results within minutes, compared with the hours required for centralized laboratory workflows, thereby enabling real‐time therapeutic adjustments that conventional systems rarely permit. In addition, PoC MS is a compact, battery‐powered, all‐in‐one device requiring no external pumps, compressed gas, or mains power. It can be operated by non‐specialist staff and consumes only microliter volumes of solvent, eliminating the need for chromatographic columns, high‐purity nitrogen, and large energy inputs, in line with green‐chemistry principles.

Beyond VPA monitoring, the versatility of PoC MS holds promise for broader therapeutic drug monitoring applications. With appropriate optimization of ionization conditions, internal standards, and SWIFT parameters, the system could be readily adapted for other antiepileptic drugs (e.g., levetiracetam, phenytoin) or drugs from different therapeutic domains. Its combination of direct whole‐blood analysis and rapid result delivery makes it particularly suited for critical care settings, such as intensive care units, emergency departments, and intraoperative environments, where timely drug‐level data can directly impact clinical outcomes. Integration with clinical decision‐support tools, including machine learning‐driven pharmacokinetic models [35], could further enhance individualized therapy and precision medicine initiatives in neurology and beyond.

This study has several limitations. First, validation was performed in a single center with a modest sample size. Larger multicenter studies with expanded cohorts are needed to confirm generalizability. Second, analytical variability increases at extremely low concentrations because PoC MS omits chromatographic separation, so residual blood‐matrix components exert a larger influence on ionization when the analyte signal approaches the noise threshold. Although such values are clinically interpreted as sub‐therapeutic and do not affect dosing decisions, future work will investigate on‐cartridge micro‐extraction, matrix‐matched calibration, and multi‐isotope internal standards to reduce matrix effects and improve precision at the lower end of the quantification range. Finally, compared with established laboratory assays, PoC MS has not yet been widely validated in routine clinical workflows, and additional studies are required to demonstrate reliability and build clinical acceptance. As the technology matures and gains wider use, user familiarity should increase and per‐test costs should decline, mirroring the adoption trajectory of bedside glucometers and ultimately enabling faster, more individualized antiseizure management for patients.

## 5 Conclusion

This study introduces a well‐designed PoC MS system for real‐time VPA monitoring in neurosurgical patients, enabling rapid and accurate quantification directly from whole blood. The system demonstrated performance comparable to conventional methods while significantly reducing turnaround time, thereby facilitating personalized clinical management.

## Author Contributions

Y.M., W.H., Z.O., and W.Z. were responsible for the study's conceptualization, framework, and design; X.F. handled blood sampling and contributed to data collection, alongside J.W. and Y.H.; X.F., J.W., Y.H., and J.B. were involved in data analysis. All authors contributed to manuscript writing, critically reviewed the manuscript, and approved the final version.

## Conflicts of Interest

Z.O. is the founder of PURSPEC Technology that is developing commercial systems for lipidomic analysis. J.B., J.W., and Y.H. are employees at PURSPEC Technology.

## Supporting information


**Table S1.** Calibration curve parameters of VPA in whole blood and serum matrices.
**Table S2.** Paired analysis of m/z 143/149 signal ratios and VPA concentrations in matched whole blood and serum matrices (*n* = 12).
**Table S3.** Method comparison of VPA quantification in 50 clinical samples with triplicate replicates using LC–MS, EMIT, and PoC MS.
**Table S4.** PoC MS monitoring of perioperative valproic acid concentrations in 9 patients.

## Data Availability

The data that support the findings of this study are available on request from the corresponding author. The data are not publicly available due to privacy or ethical restrictions.
