# Supplementary material for: Real‐Time Monitoring of the Antiseizure Drug Valproic Acid Using a Novel Point‐Of‐Care Mass Spectrometry
Source: CNS Neurosci Ther. 2025 Jul 3;31(7):e70499. doi: 10.1111/cns.70499 (PMC12223405; doi:10.1111/cns.70499)
Supplement: Supplementary file 1 — Table S1. Calibration curve parameters of VPA in whole blood and serum matrices. Table S2. Paired analysis of m/z 143/149 signal ratios and VPA concentrations in matched whole blood and serum matrices (n = 12). Table S3. Method comparison of VPA quantification in 50 clinical samples with triplicate replicates using LC–MS, EMIT, and PoC MS. Table S4. PoC MS monitoring of perioperative valproic acid concentrations in 9 patients. [file CNS-31-e70499-s001.docx]

**Supplemental Material**

**Real-Time Monitoring of the Antiseizure Drug Valproic Acid Using a Novel Point-of-Care Mass Spectrometry**

Xinqi Fang^1,2,3,4^, Junhan Wu^5^, Yuan Hong^5^, Jiexun Bu^6^, Wenpeng Zhang^7^, Zheng Ouyang^7^, Wei Hua^1,2,3,4, *^, Ying Mao^1,2,3,4, *^

1. Department of Neurosurgery, Huashan Hospital, Shanghai Medical College, Fudan University, Shanghai, 200040, China

2. National Center for Neurological Disorders, Shanghai, 200040, China

3. Shanghai Key Laboratory of Brain Function and Restoration and Neural Regeneration, Shanghai, 200040, China

4. Neurosurgical Institute of Fudan University, Shanghai, 200040, China

5. PURSPEC Technology (China) Ltd., Suzhou, Jiangsu, China.

6. PURSPEC Technology (Beijing) Ltd., Beijing, Beijing, China.

7. State Key Laboratory of Precision Measurement Technology and Instruments, Department of Precision Instrument, Tsinghua University, Beijing 100084, China.

* Correspondence to:

Mao Ying

Department of Neurosurgery, Huashan Hospital, Shanghai Medical College, Fudan University, Shanghai, 200040, China;

E-mail address: maoying@fudan.edu.cn

Hua Wei

Department of Neurosurgery, Huashan Hospital, Shanghai Medical College, Fudan University, Shanghai, 200040, China;

E-mail address: drhuawei@fudan.edu.cn

**Supplementary Tables**

**Supplementary Table 1.** Calibration curve parameters of VPA in whole blood and serum matrices.

| **Whole blood matrix** | | | | | | | | | |
| --- | --- | --- | --- | --- | --- | --- | --- | --- | --- |
| **Standard (μg/mL)** | **Replicates (n = 3)** | **m/z 143** | **m/z 149** | **ratio** | **AVE** | **SD** | **RSD** | **Measured (μg/mL)** | **Relative Error** |
| 0 | 1 | 611.930 | 6854.610 | 0.089 | 0.073 | 0.015 | 0.204 | 0.212 | / |
|  | 2 | 830.400 | 13723.180 | 0.061 |  |  |  |  |  |
|  | 3 | 1026.310 | 14984.430 | 0.068 |  |  |  |  |  |
| 10 | 1 | 891.130 | 6731.690 | 0.132 | 0.127 | 0.015 | 0.119 | 11.156 | 0.116 |
|  | 2 | 756.650 | 5422.890 | 0.140 |  |  |  |  |  |
|  | 3 | 1220.020 | 11038.290 | 0.111 |  |  |  |  |  |
| 24 | 1 | 1608.390 | 8415.490 | 0.191 | 0.193 | 0.015 | 0.079 | 24.333 | 0.014 |
|  | 2 | 1384.970 | 7719.620 | 0.179 |  |  |  |  |  |
|  | 3 | 1531.900 | 7310.120 | 0.210 |  |  |  |  |  |
| 38 | 1 | 1058.340 | 4896.230 | 0.216 | 0.226 | 0.009 | 0.041 | 30.910 | -0.187 |
|  | 2 | 1047.080 | 4594.050 | 0.228 |  |  |  |  |  |
|  | 3 | 1641.800 | 6996.200 | 0.235 |  |  |  |  |  |
| 66 | 1 | 2319.490 | 5390.230 | 0.430 | 0.446 | 0.017 | 0.039 | 74.816 | 0.134 |
|  | 2 | 2404.380 | 5433.250 | 0.443 |  |  |  |  |  |
|  | 3 | 3785.680 | 8149.980 | 0.465 |  |  |  |  |  |
| 80 | 1 | 6251.830 | 13682.010 | 0.457 | 0.453 | 0.005 | 0.011 | 76.184 | -0.048 |
|  | 2 | 4171.970 | 9191.980 | 0.454 |  |  |  |  |  |
|  | 3 | 5632.620 | 12599.660 | 0.447 |  |  |  |  |  |
| 122 | 1 | 3794.240 | 5551.630 | 0.683 | 0.679 | 0.004 | 0.006 | 121.403 | -0.005 |
|  | 2 | 3457.280 | 5119.250 | 0.675 |  |  |  |  |  |
|  | 3 | 2996.260 | 4423.500 | 0.677 |  |  |  |  |  |
| 150 | 1 | 4192.700 | 5294.540 | 0.792 | 0.816 | 0.036 | 0.044 | 148.894 | -0.007 |
|  | 2 | 3058.780 | 3827.060 | 0.799 |  |  |  |  |  |
|  | 3 | 4240.670 | 4946.110 | 0.857 |  |  |  |  |  |
| **Serum matrix** | | | | | | | | | |
| **Standard (μg/mL)** | **Replicates (n = 3)** | **m/z 143** | **m/z 149** | **ratio** | **AVE** | **SD** | **RSD** | **Measured (μg/mL)** | **Relative Error** |
| 0 | 1 | 694.390 | 2842.790 | 0.244 | 0.193 | 0.048 | 0.248 | 5.077 | / |
|  | 2 | 606.230 | 3307.220 | 0.183 |  |  |  |  |  |
|  | 3 | 866.960 | 5770.930 | 0.150 |  |  |  |  |  |
| 10 | 1 | 815.720 | 4101.130 | 0.199 | 0.211 | 0.011 | 0.053 | 8.637 | -0.136 |
|  | 2 | 810.570 | 3671.780 | 0.221 |  |  |  |  |  |
|  | 3 | 1158.460 | 5421.500 | 0.214 |  |  |  |  |  |
| 24 | 1 | 731.740 | 2580.380 | 0.284 | 0.300 | 0.043 | 0.142 | 25.652 | 0.069 |
|  | 2 | 789.120 | 2951.410 | 0.267 |  |  |  |  |  |
|  | 3 | 1080.870 | 3107.480 | 0.348 |  |  |  |  |  |
| 38 | 1 | 1244.130 | 4121.310 | 0.302 | 0.339 | 0.033 | 0.097 | 33.227 | -0.126 |
|  | 2 | 1296.250 | 3693.560 | 0.351 |  |  |  |  |  |
|  | 3 | 1930.700 | 5302.360 | 0.364 |  |  |  |  |  |
| 66 | 1 | 2087.530 | 4448.230 | 0.469 | 0.481 | 0.015 | 0.031 | 60.488 | -0.084 |
|  | 2 | 2567.970 | 5160.860 | 0.498 |  |  |  |  |  |
|  | 3 | 1931.650 | 4063.820 | 0.475 |  |  |  |  |  |
| 80 | 1 | 2194.450 | 3610.890 | 0.608 | 0.601 | 0.029 | 0.048 | 83.688 | 0.046 |
|  | 2 | 1818.560 | 2902.330 | 0.627 |  |  |  |  |  |
|  | 3 | 2504.840 | 4395.840 | 0.570 |  |  |  |  |  |
| 122 | 1 | 2721.930 | 3337.590 | 0.816 | 0.825 | 0.018 | 0.022 | 126.629 | 0.038 |
|  | 2 | 3401.320 | 4021.650 | 0.846 |  |  |  |  |  |
|  | 3 | 3454.380 | 4250.370 | 0.813 |  |  |  |  |  |
| 150 | 1 | 4026.350 | 4265.590 | 0.944 | 0.947 | 0.076 | 0.080 | 150.190 | 0.001 |
|  | 2 | 6004.390 | 5859.690 | 1.025 |  |  |  |  |  |
|  | 3 | 4336.560 | 4967.660 | 0.873 |  |  |  |  |  |

*AVE, average; SD, standard deviation; RSD, relative standard deviation;* *m/z 143: Deprotonated valproic acid (VPA) [M-H]⁻; m/z 149: Deuterated internal standard (VPA-D6) [M-D]⁻.*

**Supplementary Table 2.** Paired analysis of m/z 143/149 signal ratios and VPA concentrations in matched whole blood and serum matrices (n=12).

| **No.** | **Sex** | **Age** | **Diagnosis** | **m/z 143/149 (Whole Blood)** | **VPA Conc. in WB (μg/mL)** | **m/z 143/149 (Serum)** | **VPA Conc. in Serum (μg/mL)** |
| --- | --- | --- | --- | --- | --- | --- | --- |
| 1 | Female | 45 | Oligodendroglioma | 0.424 | 70.725 | 0.559 | 74.889 |
| 2 | Male | 44 | Astrocytoma | 0.435 | 72.908 | 0.638 | 89.903 |
| 3 | Male | 39 | Glioblastoma | 0.308 | 47.559 | 0.448 | 53.859 |
| 4 | Female | 53 | Astrocytoma | 0.241 | 34.233 | 0.364 | 37.850 |
| 5 | Female | 38 | Oligodendroglioma | 0.206 | 27.203 | 0.309 | 27.417 |
| 6 | Male | 57 | Oligodendroglioma | 0.315 | 48.892 | 0.429 | 50.275 |
| 7 | Male | 50 | Oligodendroglioma | 0.271 | 40.163 | 0.408 | 46.258 |
| 8 | Male | 25 | Astrocytoma | 0.293 | 44.568 | 0.460 | 56.135 |
| 9 | Female | 74 | Glioblastoma | 0.703 | 126.365 | 1.012 | 160.836 |
| 10 | Male | 41 | Astrocytoma | 0.518 | 89.519 | 0.699 | 101.476 |
| 11 | Male | 60 | Glioblastoma | 0.410 | 67.845 | 0.564 | 75.829 |
| 12 | Male | 62 | Glioblastoma | 0.328 | 51.570 | 0.514 | 66.355 |

*VPA, Valproic acid; WB, Whole Blood; Conc., concentration.* *m/z 143: VPA deprotonated molecule [M-H]⁻; m/z 149: [²H₆]-VPA (VPA-D6) [M-D]⁻.*

**Supplementary Table 3.** Method comparison of VPA quantification in 50 clinical samples with triplicate replicates using LC-MS, EMIT, and PoC MS.

| **No.** | **Sex** | **Age** | **Diagnosis** | **LC-MS (μg/mL)** | **Replicate** | **EMIT (μg/mL)** | **PoC MS (μg/mL)** |
| --- | --- | --- | --- | --- | --- | --- | --- |
| 1 | Male | 36 | Oligodendroglioma | 77.00 | 1 | 76 | 73.42 |
|  |  |  |  |  | 2 | 82 | 85.48 |
|  |  |  |  |  | 3 | 76 | 77.30 |
| 2 | Male | 59 | Oligodendroglioma | 60.20 | 1 | 63 | 65.52 |
|  |  |  |  |  | 2 | 62 | 61.46 |
|  |  |  |  |  | 3 | 70 | 65.68 |
| 3 | Female | 35 | Oligodendroglioma | 115.00 | 1 | 116 | 136.27 |
|  |  |  |  |  | 2 | 117 | 121.84 |
|  |  |  |  |  | 3 | 121 | 122.44 |
| 4 | Male | 47 | Oligodendroglioma | 47.10 | 1 | 46 | 45.29 |
|  |  |  |  |  | 2 | 46 | 46.92 |
|  |  |  |  |  | 3 | 50 | 42.82 |
| 5 | Female | 54 | Oligodendroglioma | 101.00 | 1 | 101 | 109.04 |
|  |  |  |  |  | 2 | 100 | 108.88 |
|  |  |  |  |  | 3 | 103 | 103.45 |
| 6 | Male | 44 | Oligodendroglioma | 87.20 | 1 | 89 | 91.46 |
|  |  |  |  |  | 2 | 93 | 88.91 |
|  |  |  |  |  | 3 | 91 | 92.98 |
| 7 | Female | 50 | Oligodendroglioma | 65.30 | 1 | 68 | 69.26 |
|  |  |  |  |  | 2 | 69 | 69.19 |
|  |  |  |  |  | 3 | 70 | 64.71 |
| 8 | Male | 51 | Astrocytoma | 73.80 | 1 | 81 | 80.95 |
|  |  |  |  |  | 2 | 82 | 83.27 |
|  |  |  |  |  | 3 | 80 | 87.46 |
| 9 | Male | 42 | Glioblastoma | 43.30 | 1 | 44 | 43.35 |
|  |  |  |  |  | 2 | 48 | 41.38 |
|  |  |  |  |  | 3 | 43 | 42.89 |
| 10 | Female | 56 | Glioblastoma | 86.40 | 1 | 84 | 90.94 |
|  |  |  |  |  | 2 | 90 | 92.70 |
|  |  |  |  |  | 3 | 94 | 97.38 |
| 11 | Female | 34 | Glioblastoma | 78.60 | 1 | 76 | 81.40 |
|  |  |  |  |  | 2 | 77 | 80.11 |
|  |  |  |  |  | 3 | 74 | 80.29 |
| 12 | Female | 26 | Oligodendroglioma | 13.90 | 1 | 20 | 12.15 |
|  |  |  |  |  | 2 | 20 | 8.33 |
|  |  |  |  |  | 3 | 19 | 10.74 |
| 13 | Female | 34 | Astrocytoma | 91.00 | 1 | 92 | 91.83 |
|  |  |  |  |  | 2 | 87 | 95.63 |
|  |  |  |  |  | 3 | 91 | 100.75 |
| 14 | Female | 44 | Astrocytoma | 23.00 | 1 | 33 | 22.49 |
|  |  |  |  |  | 2 | 31 | 21.35 |
|  |  |  |  |  | 3 | 30 | 24.60 |
| 15 | Female | 55 | Oligodendroglioma | 57.80 | 1 | 60 | 54.10 |
|  |  |  |  |  | 2 | 60 | 50.52 |
|  |  |  |  |  | 3 | 57 | 62.02 |
| 16 | Female | 69 | Glioblastoma | 52.00 | 1 | 56 | 58.72 |
|  |  |  |  |  | 2 | 52 | 57.35 |
|  |  |  |  |  | 3 | 52 | 58.51 |
| 17 | Male | 36 | Oligodendroglioma | 84.30 | 1 | 88 | 94.66 |
|  |  |  |  |  | 2 | 88 | 97.78 |
|  |  |  |  |  | 3 | 89 | 89.03 |
| 18 | Male | 45 | Glioblastoma | 60.00 | 1 | 70 | 64.13 |
|  |  |  |  |  | 2 | 66 | 57.23 |
|  |  |  |  |  | 3 | 67 | 67.56 |
| 19 | Male | 66 | Glioblastoma | 35.90 | 1 | 38 | 35.05 |
|  |  |  |  |  | 2 | 38 | 30.61 |
|  |  |  |  |  | 3 | 38 | 29.56 |
| 20 | Male | 64 | Glioblastoma | 78.80 | 1 | 86 | 79.05 |
|  |  |  |  |  | 2 | 79 | 76.66 |
|  |  |  |  |  | 3 | 82 | 77.25 |
| 21 | Male | 42 | Glioblastoma | 74.30 | 1 | 77 | 76.64 |
|  |  |  |  |  | 2 | 74 | 70.82 |
|  |  |  |  |  | 3 | 75 | 75.62 |
| 22 | Male | 56 | Glioblastoma | 64.80 | 1 | 71 | 67.51 |
|  |  |  |  |  | 2 | 65 | 57.34 |
|  |  |  |  |  | 3 | 68 | 62.27 |
| 23 | Male | 52 | Oligodendroglioma | 94.30 | 1 | 105 | 100.78 |
|  |  |  |  |  | 2 | 93 | 100.21 |
|  |  |  |  |  | 3 | 97 | 94.61 |
| 24 | Female | 34 | Astrocytoma | 60.30 | 1 | 60 | 66.66 |
|  |  |  |  |  | 2 | 58 | 65.76 |
|  |  |  |  |  | 3 | 60 | 57.69 |
| 25 | Male | 38 | Glioblastoma | 50.20 | 1 | 57 | 52.02 |
|  |  |  |  |  | 2 | 55 | 52.72 |
|  |  |  |  |  | 3 | 56 | 51.90 |
| 26 | Female | 61 | Glioblastoma | 38.50 | 1 | 41 | 37.87 |
|  |  |  |  |  | 2 | 38 | 29.02 |
|  |  |  |  |  | 3 | 40 | 32.37 |
| 27 | Female | 28 | Glioblastoma | 41.00 | 1 | 41 | 40.68 |
|  |  |  |  |  | 2 | 43 | 37.37 |
|  |  |  |  |  | 3 | 40 | 39.16 |
| 28 | Male | 50 | Glioblastoma | 57.00 | 1 | 65 | 59.91 |
|  |  |  |  |  | 2 | 62 | 58.52 |
|  |  |  |  |  | 3 | 59 | 61.42 |
| 29 | Male | 47 | Glioblastoma | 66.70 | 1 | 76 | 72.52 |
|  |  |  |  |  | 2 | 74 | 74.58 |
|  |  |  |  |  | 3 | 73 | 76.56 |
| 30 | Male | 42 | Astrocytoma | 60.10 | 1 | 66 | 62.44 |
|  |  |  |  |  | 2 | 66 | 67.83 |
|  |  |  |  |  | 3 | 63 | 64.76 |
| 31 | Male | 47 | Oligodendroglioma | 58.10 | 1 | 60 | 55.96 |
|  |  |  |  |  | 2 | 60 | 55.21 |
|  |  |  |  |  | 3 | 62 | 48.66 |
| 32 | Female | 32 | Oligodendroglioma | 65.70 | 1 | 73 | 67.22 |
|  |  |  |  |  | 2 | 74 | 74.80 |
|  |  |  |  |  | 3 | 68 | 75.47 |
| 33 | Female | 28 | Oligodendroglioma | 118.00 | 1 | 124 | 121.53 |
|  |  |  |  |  | 2 | 122 | 114.17 |
|  |  |  |  |  | 3 | 123 | 128.54 |
| 34 | Female | 32 | Astrocytoma | 49.30 | 1 | 54 | 59.92 |
|  |  |  |  |  | 2 | 53 | 50.40 |
|  |  |  |  |  | 3 | 52 | 43.52 |
| 35 | Male | 30 | Glioblastoma | 39.30 | 1 | 44 | 37.64 |
|  |  |  |  |  | 2 | 39 | 40.36 |
|  |  |  |  |  | 3 | 39 | 37.94 |
| 36 | Male | 66 | Glioblastoma | 12.60 | 1 | 19 | 10.89 |
|  |  |  |  |  | 2 | 17 | 6.78 |
|  |  |  |  |  | 3 | 19 | 8.90 |
| 37 | Male | 70 | Glioblastoma | 53.00 | 1 | 54 | 54.26 |
|  |  |  |  |  | 2 | 51 | 59.39 |
|  |  |  |  |  | 3 | 49 | 58.54 |
| 38 | Male | 33 | Glioblastoma | 60.70 | 1 | 66 | 61.83 |
|  |  |  |  |  | 2 | 65 | 56.29 |
|  |  |  |  |  | 3 | 66 | 63.41 |
| 39 | Female | 32 | Glioblastoma | 80.80 | 1 | 84 | 86.35 |
|  |  |  |  |  | 2 | 80 | 88.35 |
|  |  |  |  |  | 3 | 81 | 83.74 |
| 40 | Male | 50 | Oligodendroglioma | 53.90 | 1 | 56 | 47.80 |
|  |  |  |  |  | 2 | 54 | 54.87 |
|  |  |  |  |  | 3 | 53 | 45.02 |
| 41 | Male | 53 | Oligodendroglioma | 25.60 | 1 | 32 | 9.59 |
|  |  |  |  |  | 2 | 32 | 23.78 |
|  |  |  |  |  | 3 | 32 | 20.65 |
| 42 | Male | 43 | Oligodendroglioma | 91.10 | 1 | 99 | 109.47 |
|  |  |  |  |  | 2 | 97 | 99.76 |
|  |  |  |  |  | 3 | 92 | 102.96 |
| 43 | Male | 37 | Oligodendroglioma | 55.30 | 1 | 56 | 72.02 |
|  |  |  |  |  | 2 | 57 | 56.94 |
|  |  |  |  |  | 3 | 52 | 53.68 |
| 44 | Male | 36 | Astrocytoma | 70.80 | 1 | 69 | 73.31 |
|  |  |  |  |  | 2 | 67 | 74.98 |
|  |  |  |  |  | 3 | 65 | 65.32 |
| 45 | Male | 48 | Astrocytoma | 45.20 | 1 | 47 | 47.84 |
|  |  |  |  |  | 2 | 45 | 53.97 |
|  |  |  |  |  | 3 | 45 | 39.29 |
| 46 | Male | 50 | Astrocytoma | 85.90 | 1 | 87 | 84.78 |
|  |  |  |  |  | 2 | 89 | 81.72 |
|  |  |  |  |  | 3 | 83 | 89.43 |
| 47 | Female | 43 | Astrocytoma | 54.00 | 1 | 54 | 52.81 |
|  |  |  |  |  | 2 | 56 | 49.35 |
|  |  |  |  |  | 3 | 52 | 51.11 |
| 48 | Female | 63 | Glioblastoma | 129.00 | 1 | 131 | 126.63 |
|  |  |  |  |  | 2 | 136 | 131.30 |
|  |  |  |  |  | 3 | 130 | 124.41 |
| 49 | Male | 43 | Astrocytoma | 48.20 | 1 | 49 | 50.80 |
|  |  |  |  |  | 2 | 50 | 48.08 |
|  |  |  |  |  | 3 | 48 | 45.72 |
| 50 | Male | 68 | Glioblastoma | 75.70 | 1 | 79 | 64.33 |
|  |  |  |  |  | 2 | 77 | 80.13 |
|  |  |  |  |  | 3 | 77 | 79.14 |

*LC-MS, Liquid Chromatography-Mass Spectrometry; PoC-MS, Point-of-Care Mass Spectrometry; EMIT, Enzyme Multiplied Immunoassay Technique. LC-MS served as the reference method with single measurements; EMIT and PoC-MS data are triplicates.*

**Supplementary Table 4.** PoC MS monitoring of perioperative valproic acid concentrations in 9 patients.

| **Case No.** | **Sex** | **Age** | **Diagnosis** | **Adm** | **Pre-op** | **Post-op** | **POD7** | **Discharge** |
| --- | --- | --- | --- | --- | --- | --- | --- | --- |
| 1 | Male | 48 | Astrocytoma | 43.16 | 45.01 | 49.54 | 55.16 | 49.96 |
| 2 | Female | 45 | Oligodendroglioma | 45.99 | 56.2 | 60.56 | 66.34 | 56.03 |
| 3 | Male | 64 | Oligodendroglioma | 80.19 | 86.74 | 104.56 | 99.36 | 75.01 |
| 4 | Female | 30 | Astrocytoma | 23.24 | 24.75 | 46.52 | 51.48 | 54.47 |
| 5 | Female | 79 | Glioblastoma | 81.93 | 88.24 | 79.33 | 88.57 | 82.50 |
| 6 | Female | 54 | Glioblastoma | 88.92 | 90.04 | 119.96 | 86.41 | 87.37 |
| 7 | Male | 24 | Astrocytoma | 106.15 | 106.58 | 113.59 | 128.03 | 124.68 |
| 8 | Female | 58 | Oligodendroglioma | 24.74 | 37.97 | 9.32 | 64.68 | 70.50 |
| 9 | Male | 68 | Glioblastoma | 60.82 | 63.55 | 136.63 | 55.75 | 11.53 |

*VPA concentrations (µg/mL) measured at five perioperative time points. Adm = within 3 days of admission; Pre-op = preoperative; Post-op = within 3 days after surgery; POD7 = postoperative day 7; Discharge = prior to hospital discharge.*
